# Efficacy and safety of inhaled formoterol 4.5 and 9 μg twice daily in Japanese and European COPD patients: Phase III study results

**DOI:** 10.1186/1471-2466-11-51

**Published:** 2011-11-15

**Authors:** Miron A Bogdan, Hisamichi Aizawa, Yoshinosuke Fukuchi, Michiaki Mishima, Masaharu Nishimura, Masakazu Ichinose

**Affiliations:** 1 Clinica Medic Or, Calea Vitan no 106, Postcode 031298, Bucharest, Romania; 2 Kurume University, 67 Asahi-cho, Kurume-shi, Fukuoka 830-0011, Japan; 3 Juntendo University, 2-1-1 Hongo, Bunkyo-ku, Tokyo 113-0033, Tokyo, Japan; 4 Kyoto University, Yoshidakonoe-cho, Sakyo-ku, Kyoto-shi, Kyoto 606-8501, Japan; 5 Hokkaido University, Nishi 7-chome, Kita 15-jo, Kita-ku, Sapporo-shi, Hokkaido 060-8638, Japan; 6 Wakayama Medical University, 811-1 Kimiidera, Wakayama-shi, Wakayama 641-8509, Japan

## Abstract

**Background:**

This study evaluated the efficacy and safety of the long-acting β_2_-agonist formoterol in patients with moderate-to-severe COPD.

**Methods:**

This double-blind, placebo-controlled, parallel-group, multinational phase III study randomized patients ≥ 40 years of age with moderate-to-severe COPD to inhaled formoterol 4.5 or 9 μg twice daily (bid) via Turbuhaler^® ^or placebo for 12 weeks. Salbutamol 100 μg/actuation via pMDI was permitted as reliever medication. The primary outcome variable was change (ratio) from baseline to treatment period in FEV_1 _60-min post-dose.

**Results:**

613 patients received treatment (formoterol 4.5 μg n = 206; 9 μg n = 199; placebo n = 208); 539 (87.9%) male; 324 (52.9%) Japanese and 289 (47.1%) European. End of study increases in FEV_1 _60-min post-dose were significantly greater (p < 0.001 for both) with formoterol 4.5 and 9 μg bid (113% of baseline for both) than with placebo, as were all secondary outcome measures. The proportion of patients with an improvement in St George's Respiratory Questionnaire score of ≥ 4 was 50.2% for formoterol 4.5 μg (p = 0.0682 vs. placebo), 59.2% (p = 0.0004) for 9 μg, and 41.3% for placebo. Reduction in reliever medication use was significantly greater with formoterol vs. placebo (9 μg: -0.548, p < 0.001; 4.5 μg: -0.274, p = 0.027), with 9 μg being significantly superior to 4.5 μg (-0.274, p = 0.029). Formoterol was well tolerated with the incidence and type of adverse events not being different for the three groups.

**Conclusions:**

Formoterol 4.5 μg and 9 μg bid was effective and well tolerated in patients with COPD; there was no difference between formoterol doses for the primary endpoint; however, an added value of formoterol 9 μg over 4.5 μg bid was observed for some secondary endpoints.

**Trial registration:**

NCT00628862 (ClinicalTrials.gov); D5122C00001 (AstraZeneca Study code).

## Background

Chronic obstructive pulmonary disease (COPD) is a chronic, progressive respiratory disease that follows a course of declining lung function and markedly impaired quality of life, and places patients at a significantly increased risk of premature death [1,2]. The pulmonary component of the disease is characterized by airflow obstruction that is not fully reversible and is associated with symptoms of breathlessness, cough, and reduced physical exercise capacity resulting from inflammatory and destructive changes in the lungs. Exacerbations are common, particularly in severe stages of the disease, and frequently lead to hospitalization and can be life-threatening.

Bronchodilator therapy is the mainstay of pharmacotherapy for COPD with treatment with short-acting bronchodilators being recommended in patients with mild COPD, while long-acting β_2_-agonists (LABAs) and anticholinergics are added for patients with moderate to severe COPD. Despite an underlying inflammatory component to the disease, the use of LABAs for the treatment of COPD is generally advocated without concomitant inhaled corticosteroid (ICS) therapy. ICS monotherapy for COPD has shown only limited benefit in some studies [3,4], while improvements in symptom control, lung function and all-cause mortality has been reported with the combination of ICS/LABA budesonide/formoterol [5-7] and fluticasone propionate/salmeterol [8]. Consequently, COPD guidelines (Global Initiative for Chronic Obstructive Lung Disease [GOLD]) include bronchodilator therapy as the primary treatment in COPD without mandating concomitant use of anti-inflammatory therapy [9].

Some concerns have existed in the past regarding the safety of LABAs in patients with airway disease, fueled by a meta-analysis conducted by Salpeter et al [10] that suggested there was a doubling of the risk of respiratory death in COPD with the use of a LABA compared with placebo. In a more recent meta-analysis, Rodrigo and coworkers [11] came to a different conclusion to those of Salpeter and colleagues [10], reporting no increased risk of mortality in COPD patients using LABAs. The difference in these findings may partly relate to differences in trial selection [10,11].

The objective of this study was to evaluate the efficacy and safety of two doses of the LABA formoterol, 4.5 and 9 μg twice daily (bid), in Japanese and European patients with COPD.

## Methods

### Design

This was a randomized, double-blind, placebo-controlled, parallel-group, multinational (Japan, Romania, Russia and Ukraine) phase III study (Study code: D5122C00001; ClinicalTrials.gov identifier: NCT00628862) conducted at 65 centers in Japan and Europe. The study protocol was approved by the Institutional Review Board/Ethics Committee at all participating sites and the study was performed in accordance with the Declaration of Helsinki and the AstraZeneca policy on Bioethics and Human Biological Samples. All patients provided written informed consent prior to enrolment into the study.

The primary objective was to show that formoterol 4.5 and 9 μg bid for 12 weeks were superior to placebo in Japanese and European patients with COPD using forced expiratory volume in 1 second (FEV_1_) 60-min post-dose as the primary outcome variable.

The study consisted of an enrolment period for withdrawal of pre-study medication, a 2-week run-in period and a 12-week treatment period. Clinic visits took place at baseline (on completion of the 2-week run-in period), and at weeks 4, 8, and 12 of the treatment period.

### Study population

Male and female patients ≥ 40 years of age with a clinical diagnosis of COPD (post-bronchodilator FEV_1 _< 80% of predicted normal, and post-bronchodilator FEV_1_/forced vital capacity [FVC] < 70%) and current COPD symptoms were eligible for inclusion. Patients were also required to have a current or previous smoking history of ≥ 10 pack-years and a symptom score of at least 2 points (combination of breathlessness, cough, and/or night time awakenings due to symptoms; each assessed by the patients on a scale of 0-4 where 0 = no symptoms and 4 = severe symptoms) on at least 6 of the last 10 days of the run-in period.

Patients with a history and/or current clinical diagnosis of asthma were excluded from participating, as were those with a history and/or current clinical diagnosis of atopic disease such as allergic rhinitis. Additional exclusion criteria included use of an ICS within 4 weeks of the run-in period, COPD exacerbation requiring hospitalization and/or a course of antibiotics and/or systemic steroid therapy within 4 weeks of the run-in period, significant or unstable ischemic heart disease, or other relevant cardiovascular conditions, any other respiratory tract disorders or significant disease likely to place the patient at risk during the study.

### Study treatments

Patients received inhaled formoterol 4.5 or 9 μg twice daily (bid) via Turbuhaler^® ^or matching placebo for 12 weeks. Study treatments were taken at approximately the same time every morning and evening and immediately after measuring peak expiratory flow (PEF).

Salbutamol 100 μg/actuation via a pressurized metered-dose inhaler (pMDI) was available to relieve symptoms throughout the study period and patients could continue to take short-acting anticholinergics. Patients previously receiving long-acting anticholinergics were switched to short-acting anticholinergics at the start of the enrolment period. Glucocorticosteroid treatment was not permitted at any time during the study.

### Outcomes

The primary outcome variable was change (ratio) from baseline to the end of treatment period in FEV_1 _60-min post-dose. Secondary outcome variables included spirometry, diary variables, and assessment of health-related quality-of-life (HRQL). Spirometry endpoints were FVC 60-min post-dose, FEV_1 _and FVC pre-dose and 5-min post-dose. Diary variables were morning and evening PEF, COPD symptom scores (night-time awakenings due to symptoms, breathlessness, and cough), and use of salbutamol as reliever medication (measured as inhalations/day). HRQL was assessed using the St George's Respiratory Questionnaire (SGRQ).

Safety and tolerability were assessed by evaluation of the nature, incidence and severity of adverse events, clinical laboratory variables including clinical chemistry, hematology, and urinalysis, 12-lead electrocardiogram (ECG), blood pressure and pulse rate.

### Statistical analyses

Sample size selection was based on clinical data derived from a published 6-month study [12]. With a two-sided test at level 0.05 and 176 patients per treatment group, the study was determined to have 80% power to detect a difference between the treatment groups of at least 0.06 L in the change from baseline FEV_1 _value.

A last-observation-carried-forward (LOCF) approach was used to account for any missing week 6 data. The comparison of formoterol 4.5 and 9 μg bid with placebo was performed on the primary endpoint, mean change from baseline in FEV_1 _60-min post-dose, using an analysis of covariance (ANCOVA) model including country and treatment as fixed factors and the baseline value as covariate. A two-sided 5% significance level was used and 95% confidence intervals (CIs) for the mean difference between each dose of formoterol and placebo were calculated. The multiplicity of statistical tests was adjusted by a "closed testing procedure" under which the null hypothesis that 9 μg bid was equal to placebo was tested; if this null hypothesis was rejected then the 4.5 μg bid dose versus placebo was tested.

As for FEV_1_, the comparison of active treatments (formoterol 4.5 μg and 9 μg bid) with placebo for the secondary variables was performed using an ANCOVA model including country and treatment as fixed factors and the baseline value as covariate. The ANCOVA model used in the analysis was multiplicative. A two-sided 5% significance level was used and the "closed testing procedure" was applied.

The incidence of adverse events was calculated, and results from laboratory safety measurements, vital signs, and ECG, were analyzed primarily by means of descriptive statistics.

## Results

**Figure 1:**
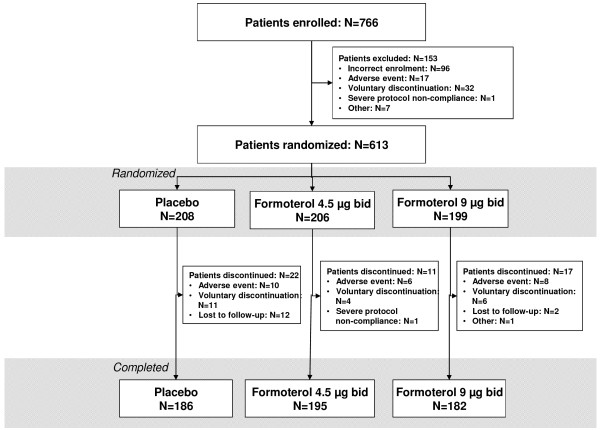
**Patients flow through the study**.

A total of 613 patients were randomized to treatment (formoterol 4.5 μg bid n = 206; 9 μg bid n = 199; placebo n = 208); 539 (87.9%) patients were male; 324 (52.9%) patients were Japanese and 289 (47.1%) were European. The mean duration of COPD since diagnosis was 4.5 years (range 0-39 years), the mean post-bronchodilator FEV_1 _was 51% of predicted normal, and the FEV_1_/FVC was 46%. Of the 613 randomized patients, 563 patients completed the study (formoterol 4.5 μg bid n = 195; 9 μg bid n = 182; placebo n = 186) and 50 patients discontinued treatment (formoterol 4.5 μg bid n = 11; 9 μg bid n = 17; placebo n = 22); the flow of patients through the study and reasons for discontinuation are shown in Figure 1. There were no major differences in baseline characteristics between the three treatment groups (Table 1).

**Table 1:** Patients' baseline characteristics by treatment group at study entry (visit 2)

	Placebo	Formoterol 4.5 μg	Formoterol 9 μg
N	208	206	199
Mean age, years (range)	66.3 (40-86)	66.7 (41-85)	67.2 (44-88)
Patients ≥ 65 years, %	59.6%	64.6%	60.8%
Male, %	89.4%	88.8%	85.4%
Japanese/European, n (%)	110/98 (52.9%/47.1%)	106/100 (51.5%/48.5%)	108/91 (54.3%/45.7%)
Mean smoking pack years (range)	47.4 (10-152)	46.1 (10-150)	46.5 (11-175)
Mean duration of disease, years (range)	4.3 (0-25)	4.2 (0-39)	4.9 (0-34)
Mean FEV_1_, L (range)^a^	1.37 (0.48-3.17)	1.30 (0.48-3.10)	1.30 (0.25-2.72)
Mean FEV_1_, % of predicted normal (range)^a^			
	52.5 (16.6-83.7)	50.4 (22.0-79.4)	51.5 (9.3-79.5)
Patients with FEV_1 _≤ 50%, %	43.3%	50.5%	48.2%
Mean FEV_1_/FVC ratio, % (range)^a^	45.6 (20.5-68.8)	44.6 (23.0-70.4)	46.5 (18.1-77.2)
Mean FEV_1 _reversibility, % (range)	11.3 (-30.3-67.2)	10.5 (-17.9-61.6)	10.7 (-57.4-63.6)

^a^Pre-bronchodilator value.

Disease-related treatment prior to enrolment included anticholinergics (55.5% of patients), inhaled selective β_2_-agonists (49.1%), xanthines (18.6%), mucolytics (16.0%), inhaled β-agonists/other drugs for obstructive airway disease (10.8%) and systemic selective β_2_-agonists (7.0%); these prior treatments were well balanced across the three treatment groups.

### Efficacy

**Figure 2:**
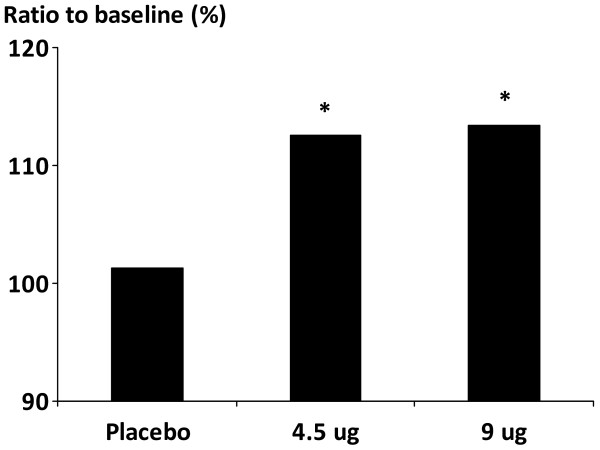
FEV_1 _at 60-min post-dose in patients with COPD receiving formoterol or placebo; *p < 0.001 vs. placebo. FEV_1 _at 60-min post-dose (ratio to baseline) in patients with COPD receiving formoterol 4.5 or 9 μg twice daily or placebo. *p < 0.001 vs. placebo.

At the end of the treatment period, increases in FEV_1 _60-min post-dose compared with baseline were significantly greater in the formoterol 4.5 and 9 μg bid groups (formoterol 4.5 μg: 112.6% of baseline; formoterol 9 μg: 113.4% of baseline; p < 0.001 for both groups) than in the placebo group (Table 2; Figure 2). No difference could be detected (0.7%) between the formoterol 4.5 μg and 9 μg bid groups (ratio of formoterol 9 μg vs. 4.5 μg 100.7%; p = 0.643)(Figure 2).

**Table 2:** Mean values at baseline and post-treatment for spirometric and other parameters

	Placebo (n = 208)	Formoterol 4.5 μg (n = 206)	Formoterol 9 μg (n = 199)
60-min post-dose FEV_1_, L			
Baseline	1.23	1.18	1.16
Post-treatment	1.25	1.33	1.31
Ratio to baseline (%)	101.3	112.6	113.4
Pre-dose FEV_1_, L			
Baseline	1.23	1.18	1.16
Post-treatment	1.24	1.23	1.21
Ratio to baseline (%)	99.8	104.5	104.7
5-min post-dose FEV_1_, L			
Baseline	1.23	1.18	1.16
Post-treatment	1.24	1.30	1.28
Ratio to baseline (%)	101.3	110.2	110.2
60-min post-dose FVC, L			
Baseline	2.77	2.71	2.61
Post-treatment	2.82	2.98	2.87
Ratio to baseline (%)	102.1	109.7	109.9
Pre-dose FVC, L			
Baseline	2.77	2.71	2.61
Post-treatment	2.80	2.81	2.70
Ratio to baseline (%)	100.7	103.6	103.4
5-min post-dose FVC, L			
Baseline	2.77	2.71	2.61
Post-treatment	2.80	2.94	2.84
Ratio to baseline (%)	101.6	108.5	108.9
Morning PEF, L/min			
Run-in	223.8	211.9	215.0
Treatment	227.6	228.1	233.5
Change from run-in	3.6	16.3	18.3
Evening PEF, L/min			
Run-in	233.8	221.4	221.9
Treatment	236.5	234.6	237.5
Change from run-in	2.4	13.2	15.8
Night-time awakening, score/day			
Run-in	0.73	0.66	0.83
Treatment	0.68	0.53	0.66
Change from run-in	-0.05	-0.13	-0.17
Breathlessness, score/day			
Run-in	1.65	1.51	1.72
Treatment	1.38	1.11	1.28
Change from run-in	-0.26	-0.41	-0.45
Cough, score/day			
Run-in	1.46	1.44	1.63
Treatment	1.26	1.11	1.21
Change from run-in	-0.20	-0.33	-0.41
Total symptom score			
Run-in	3.84	3.61	4.19
Treatment	3.32	2.75	3.14
Change from run-in	-0.51	-0.86	-1.04
Use of salbutamol, inhalations/day			
Run-in	1.86	2.09	2.40
Treatment	1.63	1.52	1.50
Change from run-in	-0.23	-0.60	-0.97
SGRQ total score			
Baseline	44.9	43.4	44.0
Last available score	42.9	38.2	38.2
Change from baseline	-2.0	-5.5	-6.4

**Figure 3:**
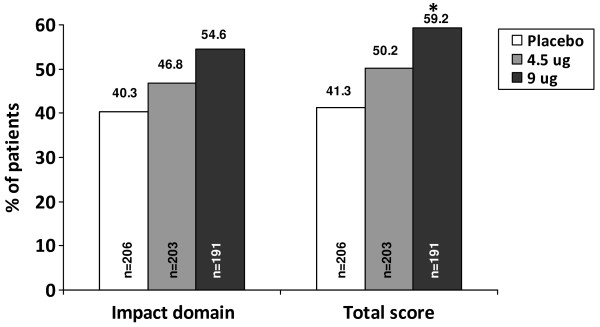
Percentage of patients with a SGRQ improvement rate > 4; *p < 0.001 vs. placebo.

Significantly greater (p < 0.05) improvements in all of the secondary outcome measures (vs. baseline) were observed with the formoterol 4.5 and 9 μg bid groups compared with those seen in the placebo group (Tables 2 and 3). While both formoterol 4.5 and 9 μg bid significantly improved the SGRQ total score compared with placebo (-3.74 and -4.45, respectively; both p ≤ 0.001), the proportion of patients with a clinically relevant improvement in SGRQ total score of > 4 units was statistically significantly greater for formoterol 9 μg bid vs. placebo (59.2% vs. 41.3%; p < 0.001) but not formoterol 4.5 μg bid vs. placebo (50.2% vs. 41.3%; p = 0.0682) (Figure 3). The difference between the two formoterol doses in the proportion of patients with an improvement in SGRQ total score of > 4 units approached statistical significance (p = 0.0757). A similar profile was observed when the SGRQ impact domain was evaluated separately (Figure 3).

**Table 3:** Differences between treatments (ratio for FEV_1 _and FVC, absolute values for other variables) for spirometric and other parameters

Variable	9 μg vs. Placebo (p-value)	4.5 μg vs. Placebo (p-value)	9 μg vs. 4.5 μg (p-value)
60-min post-dose FEV_1_, L	1.11 (< 0.001)	1.11 (< 0.001)	1.01 (0.643)
Pre-dose FEV_1_, L	1.04 (0.002)	1.04 (0.002)	1.00 (0.955)
5-min post-dose FEV_1_, L	1.09 (< 0.001)	1.09 (< 0.001)	1.00 (0.984)
60-min post-dose FVC, L	1.07 (< 0.001)	1.07 (< 0.001)	0.99 (0.642)
Pre-dose FVC, L	1.02 (0.135)	1.03 (0.026)	0.99 (0.483)
5-min post-dose FVC, L	1.07 (< 0.001)	1.07 (< 0.001)	1.00 (0.982)
Morning PEF, L/min	15.30 (< 0.001)	12.86 (< 0.001)	2.45 (0.360)
Evening PEF, L/min	13.78 (< 0.001)	10.85 (< 0.001)	2.93 (0.260)
Night-time awakening, score/day	-0.09 (0.038)	-0.10 (0.020)	0.01 (0.816)
Breathlessness, score/day	-0.17 (0.002)	-0.18 (0.001)	0.01 (0.822)
Cough, score/day	-0.13 (0.013)	-0.12 (0.023)	-0.01 (0.809)
Total symptom score	-0.41 (0.001)	-0.62 (0.001)	-0.25 (0.883)
Use of salbutamol, inhalations/day	-0.55 (< 0.001)	-0.27 (0.027)	-0.27 (0.029)
SGRQ total score	-4.45 (0.001)	-3.74 (0.001)	-0.71 (0.553)

**Figure 4:**
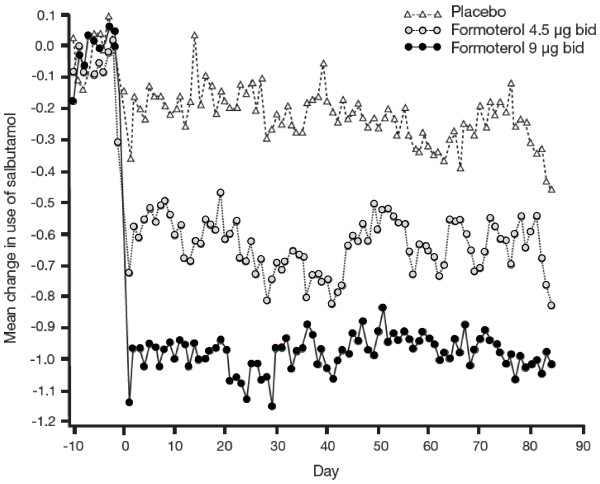
**Mean change from baseline in use of salbutamol over time**.

Similarly, while both formoterol 4.5 and 9 μg bid significantly reduced the use of salbutamol as reliever medication compared with placebo (9 μg vs. placebo difference: -0.55, p < 0.001; 4.5 μg vs. placebo difference: -0.27, p = 0.027), the reduction observed in patients receiving formoterol 9 μg bid was significantly greater than that seen in those receiving 4.5 μg bid (9 μg vs. 4.5 μg difference: -0.27, p = 0.029) (Table 3; Figure 4).

### Tolerability

Formoterol was well tolerated during 12 weeks' treatment; overall, 99 adverse events were reported by 69/206 (34%) patients receiving formoterol 4.5 μg bid, 87 adverse events by 63/199 (32%) patients receiving formoterol 9 μg bid and 100 adverse events by 69/208 (33%) patients receiving placebo. The majority of adverse events were of mild or moderate intensity and the three treatment groups displayed similar patterns of adverse events (Table 4). The most frequently reported adverse events were nasopharyngitis, COPD exacerbation, and bronchitis complication.

**Table 4:** Adverse events with an incidence > 1

Adverse event, n (%)	Placebo (n = 208)	Formoterol 4.5 μg bid (n = 206)	Formoterol 9 μg bid (n = 199)
Nasopharyngitis	20 (9.6)	24 (11.7)	25 (12.6)
COPD exacerbation	17 (8.2)	10 (4.9)	8 (4.0)
Bronchitis complication	2 (1.0)	1 (0.5)	7 (3.5)
Pneumonia	0	2 (1.0)	3 (1.5)
Ill-defined disorder	2 (1.0)	2 (1.0)	1 (0.5)
Back pain	3 (1.4)	0	1 (0.5)
Dizziness	1 (0.5)	2 (1.0)	1 (0.5)
Glucose present in urine	2 (1.0)	2 (1.0)	0

Two deaths were reported in the formoterol 4.5 μg bid group, one as a result of acute sudden cardiopulmonary failure and the second due to unknown causes (this was in a 77-year-old male; cause of death was classified as respiratory standstill); neither was considered by the investigators to be related to study treatment. Serious adverse events were reported by 4 patients receiving formoterol 4.5 μg bid, 7 patients taking formoterol 9 μg bid and 4 patients on placebo. Six patients discontinued treatment due to adverse events in the formoterol 4.5 μg bid group, 8 patients discontinued in the 9 μg bid group and 10 discontinued in the placebo group.

There were no clinically significant changes in any of the three treatment groups in mean values over time for ECG, pulse rate, blood pressure and any of the haematological, clinical chemistry and urinalysis variables.

## Discussion

The results of the current study demonstrate that formoterol at doses of 4.5 μg and 9 μg bid is significantly more effective in improving lung function (FEV_1 _60-min post-dose) in Japanese and European patients with COPD compared with placebo. The results of the secondary variables, both those related to lung function (for example, morning and evening PEF) and those related to daily diary card data (for example, COPD symptom score, and reliever medication use) and health-related quality-of-life (SGRQ total score) support the findings observed with the primary study endpoint. It is noteworthy that a clinically relevant benefit for formoterol 9 μg bid compared with 4.5 μg bid was demonstrated for the secondary endpoint of improved SGRQ score and also a statistically significant improvement for formoterol 9 μg bid vs. 4.5 μg bid for use of reliever medication. The use of reliever medication was significantly lower in the formoterol 9 μg bid group compared with 4.5 μg bid, and the proportion of patients with a clinically relevant improvement in SGRQ total score of > 4 units [13] was statistically significantly greater for formoterol 9 μg bid vs. placebo but not formoterol 4.5 μg bid vs. placebo. While these results might not be that surprising, the additional reduction in reliever medication use and increase in the proportion of patients with a clinically relevant improvement in SGRQ score might suggest that there is some additional benefit of formoterol 9 μg bid over 4.5 μg bid in this COPD patient population.

Both formoterol 4.5 and 9 μg bid for 12 weeks were well tolerated in Japanese and European patients with COPD. No clinically important safety differences between the formoterol 4.5 and 9 μg bid doses were observed. Concerns were raised regarding the ongoing safety of LABA therapy in patients with COPD following the publication of a meta-analysis of 22 studies of at least 3-month duration that suggested an increased risk of death with LABA therapy compared with placebo [10]. However, this analysis has been countered by a more recent meta-analysis which applied more rigorous study selection criteria and excluded studies with duplicate data; it also included studies of at least 1-month duration [11]. This latter meta-analysis found that LABAs reduced severe exacerbations compared with placebo (relative risk 0.78; 95% CI: 0.67-2.64) and that there was no significant difference between LABA and placebo with regard to the risk of respiratory death (relative risk 1.09; 95% CI: 0.45-2.64) [11]. The results of the current 12-week study are consistent with the latter meta-analysis; two deaths did occur in the study and although both patients had been randomized to formoterol 4.5 μg bid, neither death was considered to be related to study treatment.

Bronchodilator therapy represents the mainstay of treatment for patients with COPD. The place of LABA therapy as a bronchodilator for COPD has been revisited in recent years. LABA monotherapy is more commonly used in the management of COPD than it is in the management of asthma where LABA therapy is combined with concomitant ICS and is regarded with caution following the results of the Salmeterol Multicenter Asthma Research Trial [14], which demonstrated an increased risk of death in the salmeterol treatment arm. These data led to a black box warning being applied to both salmeterol and formoterol by the US Food and Drug Administration. Clinical studies have confirmed the efficacy of formoterol for bronchodilation in COPD in terms of improved lung function, symptoms, exacerbations, and HRQL [15-20]. Furthermore, formoterol has been shown to exert a faster onset of bronchodilatory effect compared with salmeterol in patients with COPD [21]. These data, combined with the results of the current study and the encouraging efficacy meta-analysis data described earlier [11], support the role of formoterol bronchodilator therapy in patients with COPD, with the potential caveat of excluding patients with an asthma component, as recommended in the current GOLD guidelines [9].

## Conclusion

The results of the current study confirm that formoterol at doses of 4.5 and 9 μg bid is an effective and well tolerated first-line treatment option in the management of COPD, with additional benefits evident at the higher dose in terms of a reduced need for reliever medication and improved health-related quality-of-life.

## List of abbreviations

ANCOVA: analysis of covariance; bid: twice daily; CI: confidence interval; COPD: chronic obstructive pulmonary disease; ECG: electrocardiogram; FEV_1_: forced expiratory volume in 1 second; FVC: forced vital capacity; GOLD: Global Initiative for Chronic Obstructive Lung Disease; HRQL: health-related quality-of-life; ICS: inhaled corticosteroid; LABA: long-acting β_2_-agonist; LOCF: last-observation-carried-forward; PEF: peak expiratory flow; pMDI: pressurized metered-dose inhaler; SGRQ: St George's Respiratory Questionnaire.

## Competing interests

Professor Miron Bogdan has received honoraria over the past year from Glaxo SmithKline, AstraZeneca, Pfizer and Actelion. These have not inappropriately influenced his work. Professor Bogdan has never received financial support from the tobacco industry. Professor Hisamichi Aizawa has served as a member of scientific advisory boards, received honoraria for lectures or research grants from GlaxoSmithKline KK, Nippon Boehringer Ingelheim, Novartis Pharma KK and AstraZeneca KK. Professor Yoshinosuke Fukuchi has received honoraria for lectures from GlaxoSmithKline KK, Nippon Boehringer Ingelheim, Novartis Pharma KK, Abbott Japan, Otsuka Pharmaceutical, and AstraZeneca KK. Professor Michiaki Mishima has served as a member of scientific advisory boards, received honoraria for lectures or research grants from GlaxoSmithKline KK, Nippon Boehringer Ingelheim, Pfizer Japan, MSD KK, Kyorin Pharmaceutical and AstraZeneca KK. Professor Masaharu Nishimura has served as a member of scientific advisory boards, received honoraria for lectures or research grants from GlaxoSmithKline KK, Nippon Boehringer Ingelheim, Novartis Pharma KK, Abbott Japan, Pfizer Japan, MSD KK, Kyorin Pharmaceutical and AstraZeneca KK. Professor Masakazu Ichinose has served as a member of scientific advisory boards, received honoraria for lectures or research grants from GlaxoSmithKline KK, Nippon Boehringer Ingelheim, Novartis Pharma KK, Abbott Japan and AstraZeneca KK.

The study was funded by AstraZeneca.

## Authors' contributions

MB and MI made significant contributions to the conception and design of the study. MB, HA, YF, MM, MN and MI made significant contributions to the acquisition of data, analysis and interpretation of data, and drafting of the manuscript. All authors read and approved the final manuscript.

## Pre-publication history

The pre-publication history for this paper can be accessed here:

http://www.biomedcentral.com/1471-2466/11/51/prepub
